# fNIRS in global child health research: insights from failure mode and effects analysis on a longitudinal project in Bangladesh

**DOI:** 10.1117/1.NPh.13.S1.S13011

**Published:** 2026-03-31

**Authors:** Renata Di Lorenzo, Jessica E. Anderson, Talat Shama, Eileen F. Sullivan, Navin Rahman, Charles A. Nelson

**Affiliations:** a Boston Children’s Hospital, Division of Developmental Medicine, Department of Pediatrics, Boston, Massachusetts, United States; b Harvard Medical School, Boston, Massachusetts, United States; c Boston University, Neurophotonics Center, Biomedical Engineering, Boston, Massachusetts, United States; d International Centre for Diarrhoeal Disease Research, Bangladesh; e Harvard Graduate School of Education, Cambridge, Massachusetts, United States

**Keywords:** functional near-infrared spectroscopy, global health, child brain development, failure mode and effects analysis

## Abstract

**Significance:**

Neuroimaging research on child development in low- and middle-income countries (LMICs) remains limited, in part due to substantial implementation challenges. Although functional near-infrared spectroscopy (fNIRS) is a promising tool in these contexts, its use is constrained by barriers that are not yet systematically characterized.

**Aim:**

We systematically evaluate the challenges encountered during a 3-year longitudinal fNIRS project with children in Dhaka, Bangladesh. We aim to identify the major challenges affecting data quality and collection, compare our findings with similar studies in other LMICs, and gather practical guidance for fNIRS implementation in LMICs.

**Approach:**

We applied failure mode and effects analysis to systematically identify the major challenges in the project. We also polled researchers with experience in similar fNIRS studies in other LMICs and compiled mitigation strategies.

**Results:**

High-risk challenges were primarily related to fNIRS headcap fit, onsite staff procedures, and environmentally related fNIRS equipment functionality. Most of these challenges were also reported by other polled sites. Effective mitigation strategies have been compiled based on experience in Dhaka and insights from multiple LMICs.

**Conclusions:**

We provide valuable insights into the challenges of implementing fNIRS in LMICs by identifying high-priority challenges and effective mitigation strategies, ultimately informing more equitable and reliable fNIRS research in global child health.

## 1 Introduction

Functional near-infrared spectroscopy (fNIRS) offers a non-invasive method to assess how neural metabolism is influenced by experience.^1^^–^^3^ This effort is particularly important for children growing up in low- and middle-income countries (LMICs), where exposure to both biological (e.g., malnutrition) and psychosocial (e.g., poverty) adversities is highly prevalent.^4^^,^^5^ Although the field is growing, fNIRS research in LMICs remains limited, partially due to methodological and infrastructural barriers. Identifying these challenges and finding effective ways to overcome them is crucial to advancing child development research in LMICs. This work contributes to such efforts by systematically evaluating the challenges encountered in a large longitudinal project conducted in Bangladesh, a country with persistently high rates of malnutrition and poverty.^6^ Using failure mode and effects analysis (FMEA), we present a structured framework to evaluate not only the types of challenges encountered but also their severity and the risks they pose to data collection and quality, with the goal of identifying potential solutions and recommendations. In the following introductory sections, we review the advantages and limitations of using fNIRS in developmental research, summarize the challenges reported in infant studies from LMICs, and present our framework and aims.

### 1.1 Advantages of fNIRS for Developmental Research

Neuroimaging tools have become central to developmental research, offering objective estimates of brain function without requiring active participation or verbal responses. This is particularly valuable when studying young children, whose behavioral repertoire is limited. By enabling the use of passive tasks that are minimally influenced by culture, these methods reduce dependence on language or socially specific behaviors, making them more adaptable across diverse cultural contexts than traditional behavioral assessments. Among available methods, fNIRS provides unique advantages for developmental studies across different settings. fNIRS estimates localized cortical brain activity by measuring changes in hemoglobin concentration using near-infrared light.^7^ Compared with techniques such as electroencephalography (EEG) and functional magnetic resonance imaging (fMRI), fNIRS has a superior tolerance to movement, which is particularly advantageous when working with awake children who often struggle to remain still during assessments. Moreover, from a child’s perspective, the equipment involved in performing fMRI can be intimidating due to loud noise, the need to lie still, and the requirement for physical separation from caregivers during scanning. By contrast, fNIRS uses smaller, quieter equipment and allows children to remain closer to a caregiver, thereby providing a more comfortable and less stressful testing experience. Although fNIRS has lower spatial resolution than fMRI, it is more portable and affordable, making this method easier to implement in a variety of clinical and research settings, including those with limited infrastructure and resources.

### 1.2 Common Difficulties in Developmental fNIRS Studies

Despite its benefits, fNIRS presents some difficulties in developmental research. Although fNIRS signal is less susceptible to motion artifacts than other neuroimaging techniques, such as MRI and EEG, it is still affected by participant movement.^8^^,^^9^ To mitigate this, researchers design protocols to keep young participants calm and engaged,^10^ and use appropriate processing pipelines to reduce motion artifacts from the fNIRS signal.^11^ Another key challenge is placing the headgear (e.g., headcaps and headbands) accurately and consistently, which is important because its position determines where the optodes sit on the scalp and consequently which cortical areas are measured. If placement varies across participants, it can lead to inconsistent brain coverage and more variability in the data quality, increasing the risk of errors in the results and interpretation.^12^ Precise headgear placement requires taking a few head measurements and aligning optodes (or other guide marks) to key scalp landmarks, which can be difficult in infants and toddlers due to frequent movement. Other factors that complicate the fitting process include variability in head shape during early development,^13^ morphological differences across ethnic groups,^14^^,^^15^ and certain hair characteristics (e.g., texture, volume, and hairstyle). Although most commercially available headgears allow for some flexibility, these often do not fully accommodate the range of head shapes and hair differences observed in young children, potentially compromising optode–scalp contact and overall data quality. A related limitation is the lack of MRI templates adequate for most ethnicities and age ranges. These templates are crucial for accurately mapping fNIRS signals to cortical areas when processing collected data. Although limitations in headgear fit and MRI template availability are common in developmental research globally, these are especially pronounced in LMICs, where population-specific solutions remain underdeveloped. In addition to these methodological limitations, the ability to conduct such studies with young children relies on obtaining informed consent from the primary caregiver. Caregivers may have concerns about device safety, which often stem from limited familiarity with or exposure to research studies, neuroimaging methods, or fNIRS specifically.

### 1.3 Implementing Developmental fNIRS Studies in LMICs

A growing number of research projects are including fNIRS in their study designs as a tool to investigate the relations between adversity exposure and child development in LMICs (see Arrendondo for a review^16^). However, beyond challenges common to all developmental fNIRS studies, additional context-specific barriers tend to arise in LMIC settings. It is important to recognize that LMICs are comprised of highly diverse environments, varying widely in climate, culture, infrastructure, and other factors, all of which can uniquely affect study implementation. A few key publications from rural Africa^17^^–^^19^ have described the practical issues of conducting fNIRS research in a few LMICs, including challenges related to environmental conditions, infrastructure, and local technical support. For instance, climate-related factors such as heat, high humidity, and high levels of dust can affect equipment functionality and signal quality (e.g., via corrosion of metallic parts). Infrastructure limitations, including unreliable electricity and internet access, can disrupt data collection and storage, whereas equipment transport and customs delays can further hinder timely study implementation. Finally, limited local technical support often makes it difficult to address equipment malfunctions quickly and delays data collection. The authors from past work propose several mitigation strategies to these context-specific challenges; for example, [installing air conditioning (AC) or fans to mitigate heat and humidity].

Although prior research has provided important qualitative insights into the complexities of implementing developmental fNIRS studies in few LMICs, these accounts remain largely anecdotal and descriptive. To develop effective and timely mitigation strategies, it is essential to understand not only the types of challenges encountered but also their severity and the risk they pose to data collection and quality. A structured and systematic approach to evaluating these risks can support the practical and reliable use of fNIRS across diverse LMIC settings.

### 1.4 Framework for Assessing fNIRS Implementation

One promising method for systematically identifying and addressing challenges in developmental fNIRS studies in LMICs is the use of FMEA.^20^ FMEA is a structured risk assessment tool widely adopted in industry^21^ and healthcare,^22^ for example, to reduce errors in hospital medication delivery.^23^^,^^24^ FMEA allows identification and prioritization of potential pitfalls or “failure modes” (referred to as “challenges” in this work) that pose the greatest risk of disrupting a procedure (e.g., data collection). Ultimately, this structured approach helps focus prevention and mitigation efforts where they are most needed, enhancing the overall efficiency and reliability of study implementation.

This work uses FMEA to systematically identify the major challenges associated with implementing developmental fNIRS research within an LMIC setting, specifically a densely populated urban neighborhood in Dhaka, Bangladesh. We apply FMEA to a set of procedures that are part of the Bangladesh Malnutrition Trial (BMT; ClinicalTrials.gov: NCT05629624), a longitudinal project of 1- to 3-year-old children assessing the effects of nutrition and psychosocial stimulation interventions on brain and behavioral development (for more details, see Shama et al.^25^); in this project, fNIRS data were utilized as one of the outcome measures of the intervention. The FMEA evaluation targets fNIRS-specific components of the BMT project after its completion, spanning participant recruitment, data acquisition, and online data transfer.

### 1.5 Aims of Current Work

This work has four key aims. The first goal is to identify the full range of challenges encountered in BMT and highlight those with the greatest potential impact on data quality and study implementation. Second, we compare these to findings from other LMIC-based studies to determine which challenges are common and which are unique to our setting. Third, we compile effective mitigation strategies used both in Dhaka and across other LMIC contexts. These strategies are drawn from the FMEA of the BMT project, prior experience of several authors of this work with the Bangladesh Early Adversity Neuroimaging (BEAN)^26^ project (conducted in the same location by the same local team, using fNIRS to study child development), and a poll of fNIRS researchers working in Sub-Saharan Africa,^27^^,^^28^ South America,^29^ and South Asia.^30^ Finally, this is the first work to apply FMEA to developmental fNIRS research, providing a proof of concept for its value in identifying high-risk challenges. This approach could be adopted by other research teams to inform study design, prepare for future waves of data collection, or monitor study progress, particularly in complex or low-resource settings. By applying a structured risk-assessment approach, this work aims to inform the development of more refined and context-appropriate protocols and to advance efforts toward effective fNIRS implementation in diverse low-resource settings.

## 2 Methods

### 2.1 Project Assessed

We conducted FMEA on data gathered from a 3-year, community-based, randomized controlled trial carried out in the Mirpur district of Dhaka, Bangladesh. Mirpur is a densely populated urban neighborhood in a hot, humid tropical climate with high levels of poverty and malnutrition. The local research team had an established relationship with the Mirpur community due to involvement in earlier studies in the same neighborhood, such as the BEAN project which began in 2015 and involved the use of fNIRS. This ongoing relationship had fostered trust within the community. The Dhaka team built on this foundation and led the recruitment and data collection efforts for the BMT project (see Methods S1 and S2 in the Supplementary Material, respectively), currently assessed in the FMEA. A team at Boston Children’s Hospital collaborated with the Dhaka research team and led the data quality checking, processing, and analysis efforts.

The BMT project enrolled 234 1-year-old children who were followed longitudinally over a 3-year period, and 75 3-year-old children who completed a single assessment in year 1 of the project. At baseline, both groups underwent a comprehensive battery of assessments, including neuroimaging (fNIRS and EEG), behavioral evaluations, surveys, and health-related measures. The 1-year-old group was reassessed annually at ages 2 and 3, following a similar protocol, which included fNIRS and other measures. Families received transport, healthcare, and meals to support participation throughout the project. Participants were generally scheduled through home visits by the surveillance workers and later coordinated via phone. An effort was made to allow for flexible scheduling to fit the local community, which is organized in a collectivist way and operates best with verbal communication and week of scheduling rather than via methods such as written communication and scheduling far in advance that are typical in Western settings. Further details on recruitment and procedures are provided in Methods S1 in the Supplementary Material and in Shama et al.^25^

#### 2.1.1 fNIRS apparatus and fNIRS room setup

**Fig. 1:**
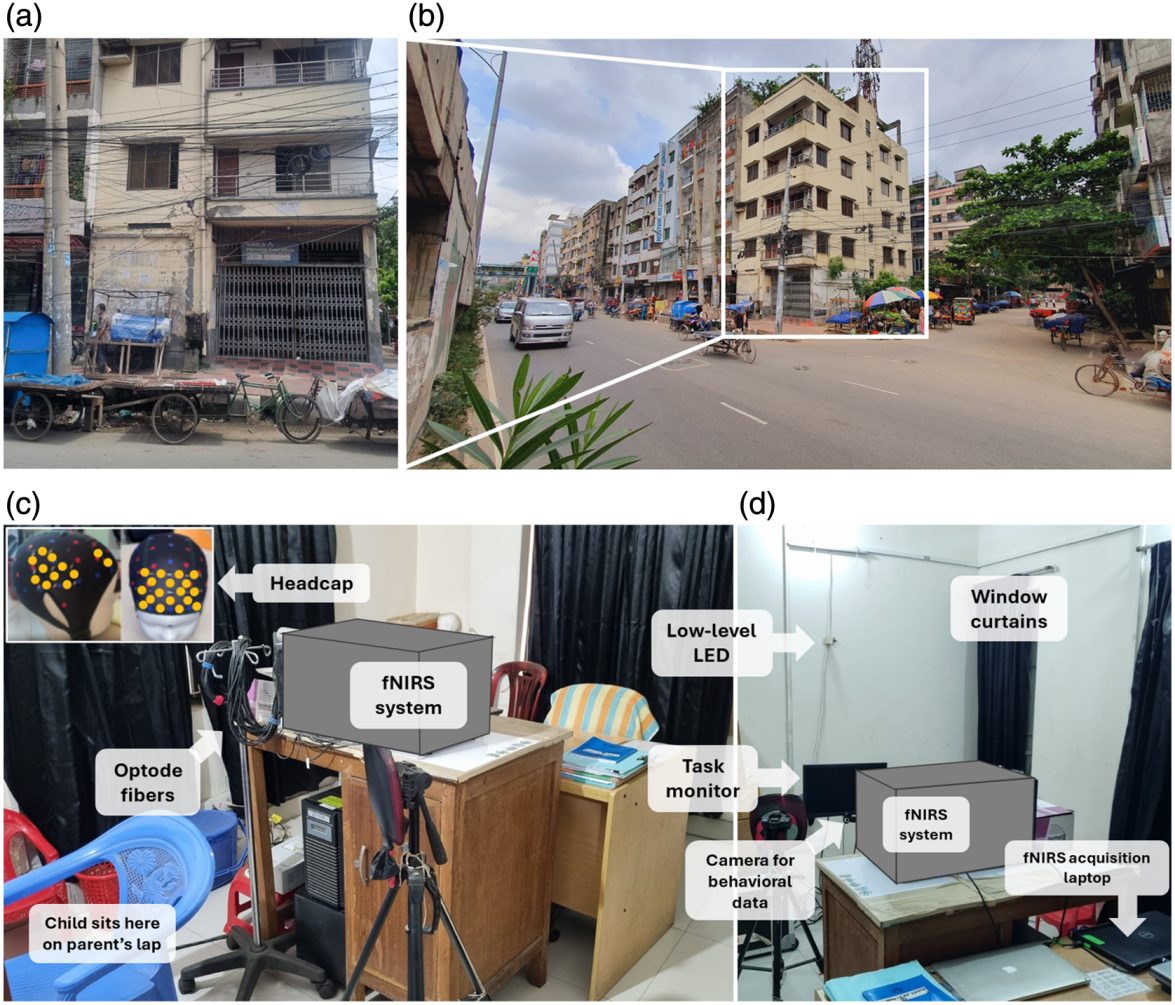
Site of data collection in Mirpur, Dhaka, Bangladesh. (a) Entrance door to the site. (b) Exterior and surroundings of the site. (c) and (d) Setup of the fNIRS testing room. (c) Setup from the front, where the child sat on the parent’s lap. (d) Setup from behind the child. Key elements of the setup are labeled. The inset photo of the headcap has yellow dots that indicate channel locations.

The BMT project employed an optical topography fNIRS system using a headcap configured for 42 channels. The fNIRS array can be seen in the inset of Fig. 1 and more fully in Fig. S1 in the Supplementary Material. The fNIRS system had been previously installed at the Mirpur clinic during BEAN with technical assistance from the manufacturer’s engineers. The room setup included one laptop dedicated to fNIRS data recording and a second laptop that ran the paradigms, transmitted visual stimuli to a monitor, played audio stimuli through speakers, and sent event markers to the recording laptop. The fNIRS system was used in the BEAN project over 5 years to test multiple cohorts of participants.^26^ Thus, the basic infrastructure was in place, and the local project team was familiar with the fNIRS technology as well as with project procedures prior to starting the BMT project. Although the fNIRS system is robust and does not typically require particular maintenance, prolonged exposure to local environmental conditions (e.g., high humidity, dust, heat, and electrical instability) contributed to system issues during BEAN’s fifth year. The system was returned to the manufacturer for repair before the start of the BMT project. Prior to BMT, the team installed an air purifier, a dehumidifier, a stable power supply, and a voltage protector in the fNIRS room to prevent future problems. An air conditioner had been in place since the beginning of BEAN. This room was devoted solely to the BEAN and BMT projects.

Additional equipment enhancements implemented before BMT data collection included the acquisition of a new monitor for stimulus display and an updated computer equipped with E-Prime 3 for running paradigms. Daily dusting of the fNIRS room was implemented as a preventative measure against dust-related issues. The fNIRS room setup is illustrated in Fig. 1. For this project, participants wore a stretchable mesh fabric headcap (see Fig. 1, inset) populated with the fNIRS optodes. Optodes were arranged in two lateral 10-channel arrays and one frontal 22-channel array as shown in Fig. S1 in the Supplementary Material. Source-detector separation ranged between 2 and 3 cm depending on headcap size. Further information on fNIRS data collection procedures can be found in Methods S2 in the Supplementary Material.

### 2.2 FMEA Methodology

FMEA is a structured risk-assessment tool used to identify challenges, analyze their causes and effects, and prioritize those with the highest negative impact to guide mitigation strategies.^20^ The current work applies FMEA retrospectively to data extracted from the BMT project, focusing only on parts of the BMT project relevant to collecting and analyzing fNIRS data. FMEA involves assembling experts with relevant experience to identify challenges, document their causes and effects, propose or report mitigations, and rate each challenge for occurrence, detection, and severity. The occurrence, detection, and severity ratings are then multiplied to calculate a risk priority number (RPN) separately for each challenge. The RPN helps researchers identify the challenges that carry the highest overall risk of disrupting a process and should therefore be prioritized for mitigation efforts. Our analysis focused on both high-risk (high RPN) and high-severity (high severity ratings) challenges. We include high-severity challenge analysis as some severe but infrequent issues may still require attention even if they have lower RPNs.

#### 2.2.1 Assembly of team of experts

The assembled team of experts, comprising six individuals, included Dhaka team members involved in BMT fNIRS data collection, team members from the Boston site dedicated to data quality checks, and experts in fNIRS signals. Two individuals were from Bangladesh and were involved in participant recruitment, data collection (including fNIRS), and data management for both the project assessed here (BMT) and the prior project BEAN.^26^ Three team members were based at the Boston partner site on the BMT project, where data quality check-ups and analyses were performed. Collectively, their expertise included fNIRS paradigm design, regular data quality and transfer check-ups with the Dhaka team, infant/toddler fNIRS data collection in low- and high-resource settings, infant fNIRS data analysis, and management of server and software infrastructure for secure data sharing among sites. A sixth expert based in Boston, external to the BMT project, joined the FMEA team due to past experience in fNIRS data collection and analysis of research in both low- and high-resource settings. A seventh individual, familiar with the project and fluent in both English and Bangla, participated in some discussions to support communication among sites. The primary methods of communication among the team of experts to accomplish the FMEA process were email and remote video calls. Importantly, the team’s combined expertise covered all the phases of the project, including local language and cultural context of the data collection site, first-hand experience with participant recruitment and data collection, and technical expertise in device operation and data processing.

#### 2.2.2 Challenge identification and assessment

**Fig. 2:**
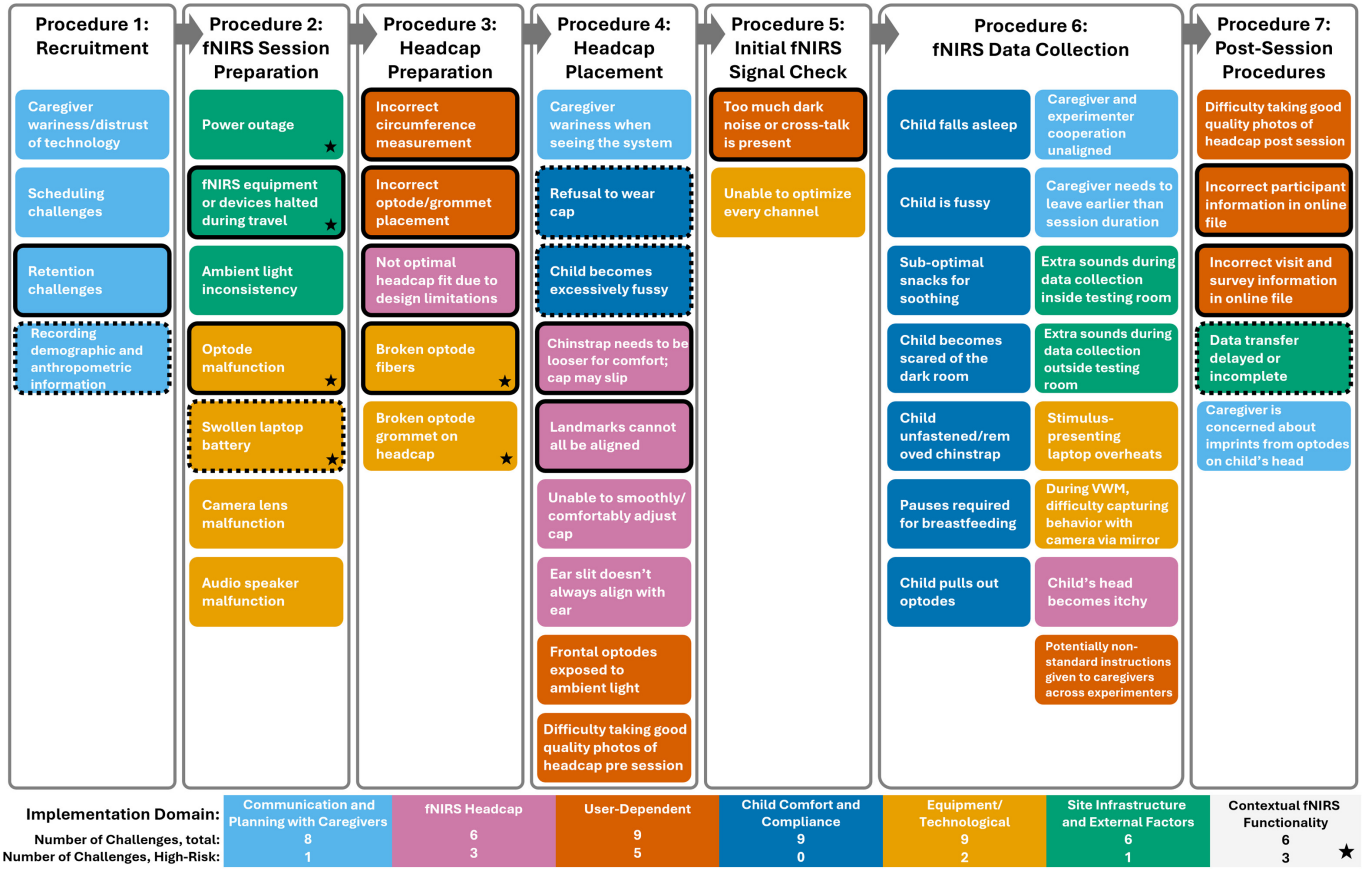
Identified challenges are presented in columns according to their assigned sequential fNIRS procedure and are color-coded by implementation domain as shown in the legend. Stars indicate the challenges that fell into the Contextual fNIRS Functionality implementation domain. Solid black borders indicate high-risk challenges identified from the FMEA. Dashed black borders indicate high-severity challenges that were not also noted as high-risk.

The team of experts identified challenges encountered within each of the following seven fNIRS procedures: recruitment, fNIRS session preparation, headcap preparation, headcap placement, initial fNIRS signal check, fNIRS data collection, and post-session procedures (see Fig. 2). For each identified challenge, the team generated possible causes, effects, and any relevant mitigation strategies.

Note that we did not assess the challenges addressed during the earlier BEAN project, as these were resolved before the start of the BMT project (the focus of this FMEA). Solutions implemented in BEAN were also used in the BMT project and reported in Sec. 2.1.1.

#### 2.2.3 Assign ratings

Our team of experts used a 1 to 10 scale to independently evaluate the detection, severity, and occurrence of each challenge, following standard FMEA guidelines. Occurrence ratings reflected how often a challenge happened, with higher scores denoting more frequent occurrence. Detection ratings assessed the chance that a challenge would go unnoticed when it occurred; higher rating scores indicated lower detectability (i.e., the challenge would almost always go undetected). Importantly, detection ratings were independent of whether the issue could be resolved given available resources. Severity ratings reflected the likelihood of a challenge to negatively impact the ability to collect data or data integrity, including effects on child comfort and security during testing, equipment functioning, data quality, and usability; higher scores indicated greater negative impact (see Methods S3 in the Supplementary Material and FMEA processes^20^^–^^24^ for further explanation). If a team member did not have expertise to provide a particular rating, then they did not assign a value. Prior to rating the challenges, each expert received detailed instructions (documented in Methods S3 in the Supplementary Material), which were reviewed and discussed as a group to clarify any questions.

A subset of challenges received three separate occurrence ratings, one for each project year, to capture potential changes in frequency over time. Such variability was anticipated due to factors such as increasing experimenter proficiency, prolonged equipment usage, and greater parental familiarity with study procedures. For example, issues with headcap preparation may have happened less often as the team became more skilled with fNIRS protocols. Yearly occurrence ratings were assigned to all challenges related to headcap preparation, headcap placement, initial fNIRS signal check, fNIRS data collection, and one post-session procedures challenge (“difficulty taking good pictures of headcap placement post session”). Challenges related to recruitment, fNIRS session preparation, and the remaining post-session procedures were rated once for the entire duration of the project.

### 2.3 Data Analysis and Cross-Context Comparison

Quantitative processing and analysis were performed using MATLAB R2024a.^31^

#### 2.3.1 Detection, severity, and occurrence average ratings

For each challenge, we calculated the average expert rating separately for detection, severity, and occurrence. For detection and severity, we calculated a single average rating across all years. For challenge occurrence, we calculated an average rating per year for challenges rated annually, resulting in three average ratings per each of those challenges. For challenges rated once across the entire project, we calculated a single average occurrence rating. Ratings that showed large disagreement (i.e., standard deviation greater than 2.0) were reviewed and revised through team discussion (see Methods S4 in the Supplementary Material for a full description of the rating consensus procedure).

#### 2.3.2 Categorization into implementation domains

To synthesize and streamline our analysis of the FMEA-identified challenges, we grouped them into seven implementation domains. These domains were established through consensus between two team experts involved in the FMEA analysis, both of whom have relevant experience implementing fNIRS studies across different research contexts and age groups. Drawing from this expertise, we generated domains suitable to cover all the identified challenges and to group them by common themes relevant to project implementation, such as challenge potential causes, mitigation approaches, the actions or equipment used when the challenge occurred, and the personnel or resources involved. The domains reflect central dimensions influencing the implementation of fNIRS research and include, as noted in Table 1 and represented in Fig. 2, the following: Communication and Planning with Caregivers, fNIRS Headcap, User-Dependent factors, Child Compliance and Comfort, Equipment/Technological factors, Site Infrastructure and External Factors, and Contextual fNIRS Functionality. The Contextual fNIRS Functionality domain was defined to isolate challenges associated with implementing fNIRS technology in LMIC settings. This domain includes a subset of challenges selected from the broader Equipment/Technological factors and Site Infrastructure and External Factors but excludes those not directly related to fNIRS functionality (e.g., malfunction of speakers and background urban noise).

**Table 1:** Description of each implementation domain. Each of the challenges identified was assigned to one of the first six domains, with a few additionally allocated to the seventh Contextual fNIRS Functionality domain (see Fig. 2). Examples of the challenges assigned to each domain are included.

Implementation Domain	Scope of Challenges
Communication and Planning with Caregivers	Ineffective communication and coordination with the child’s caregiver(s)
	For example, Scheduling challenges, Caregiver wariness/distrust of technology
fNIRS Headcap	Aspects of the headcap’s design, components, or fabric affecting optimal fit or comfort for children in BMT (excluding fNIRS optical components)
	For example, Landmarks cannot all be aligned, Child’s head becomes itchy
User-Dependent	Human error or limited experimenter’s familiarity with and insight into the procedures
	For example, Incorrect circumference measurement, Incorrect optode/grommet placement
Child Compliance and Comfort	Child behaviors and needs impacting data collection
	For example, Refusal to wear cap, Child is fussy, Child pulls out optodes
Equipment/Technological	Technical difficulties with both core fNIRS components and supporting devices
	For example, Broken optode fibers, Stimulus-presenting laptop overheats
Site Infrastructure and External Factors	Infrastructural barriers and other interferences
	For example, Ambient light inconsistency, Data transfer delayed or incomplete
Contextual fNIRS Functionality	Subset of challenges from the broader domains Equipment/Technological; Site Infrastructure and External Factors, focusing on context-specific aspects negatively impacting the use or performance of core fNIRS components
	For example, Power outage, fNIRS Equipment halted during travel, Optode malfunction

#### 2.3.3 Analysis of occurrence ratings over time

For challenges with occurrence ratings provided by project year to allow for visual and descriptive comparisons of trends over time we calculated the average occurrence score per implementation domain for each year. To statistically assess whether changes in challenge frequency over time were significant within each domain, we conducted non-parametric Wilcoxon signed-rank tests on individual challenge occurrence ratings, comparing paired years (e.g., year 1 versus year 2).

#### 2.3.4 Major challenges to prioritize: high-risk and high-severity challenges

We computed an RPN for each challenge using the standard FMEA equation: RPN = occurrence * detection * severity. For challenges with separate occurrence ratings across years 1 to 3, we averaged the yearly values to obtain a single mean RPN representing the entire 3-year project. All challenges were then ranked in descending order of RPN to identify those with the greatest potential negative impact on data quality and implementation. Following standard FMEA guidelines, high-risk challenges were defined as those falling within the top quartile of RPN values. In addition, we also ranked challenges by severity rating alone to ensure that low-frequency or easily detectable, but highly disruptive, challenges were not overlooked. Challenges in the top quartile of severity scores were flagged as high-severity.

In addition, to explore whether some implementation domains carried greater overall risk, we calculated the average RPN for each of the seven implementation domains by including all challenges assigned to each domain, regardless of their individual RPN ranking. The same approach was used to calculate average severity scores. Full results of this exploratory analysis are reported in Results S2 in the Supplementary Material.

### 2.4 Cross-Context Comparison of Challenges and Mitigation Strategies

To evaluate whether the challenges observed in BMT were context-specific or reflective of broader patterns across other LMICs, we polled researchers with experience in other neurodevelopmental fNIRS projects in LMICs. The researchers who answered our poll are key contributors to projects conducted in three distinct rural locations: Shivgarh, Uttar Pradesh, India, with children 4 months to 2 years old,^30^ Kiang regions of The Gambia with children from 1 month to 5 years old,^27^^,^^28^ and the Ecuadorian Amazon with children 3 years to 6 years old.^29^ The number of subjects for these studies ranges from as few as 42 up to several hundred. The four researchers were asked to assess whether challenges identified in the BMT project were also observed at their study sites. For each of the seven sequential fNIRS procedures (e.g., recruitment), they indicated which of the challenges they had encountered and, if applicable, described any mitigation strategies used. Researchers skipped questions on any procedures outside their area of expertise. Three researchers responded via an online survey and one participated via video call following the same protocol. Additional challenges and mitigation strategies were recorded. Two of the researchers had overlapping experience in The Gambia (both had overlapping experience with one project^28^ and one researcher had experience with an additional project^27^). Their responses were merged. From the poll results, we then compared which high-priority challenges found in the BMT were also reported by other sites, and which were specific to BMT.

We compiled the mitigation strategies identified by the FMEA process, the poll conducted, and a targeted (non-systematic) literature search of developmental fNIRS studies conducted in LMICs (further description is found in Results S3 in the Supplementary Material).

## 3 Results

### 3.1 Compiled Challenges and Ratings Consensus

The team of experts identified a total of 47 challenges across the seven fNIRS procedures as shown in Fig. 2. The number of identified challenges per procedure ranged from two (for initial fNIRS signal check) to 15 (for fNIRS data collection). The number of challenges per implementation domain ranged from six (for three of the seven domains) to nine (for another three domains). Results S1 in the Supplementary Material reports further details of the results of our consensus process and a summary of the rating values.

### 3.2 Occurrence Rating Trends over Project Years

The occurrence ratings (i.e., how often a challenge happened) which were assigned once for the full project duration (n=15 challenges) ranged from 2.0 to 5.0 (M=3.0, SE = 0.2). Of the 47 total challenges, 32 received separate occurrence ratings for each project year. The occurrence ratings which were assigned per project year decreased over time and ranged from 1.3 to 7.5 (M=4.7, SE = 0.3) for year 1, 1.7 to 6.3 (M=3.3, SE = 0.2) for year 2, and 1.0 to 6.3 (M=2.7, SE = 0.2) for year 3.

**Fig. 3:**
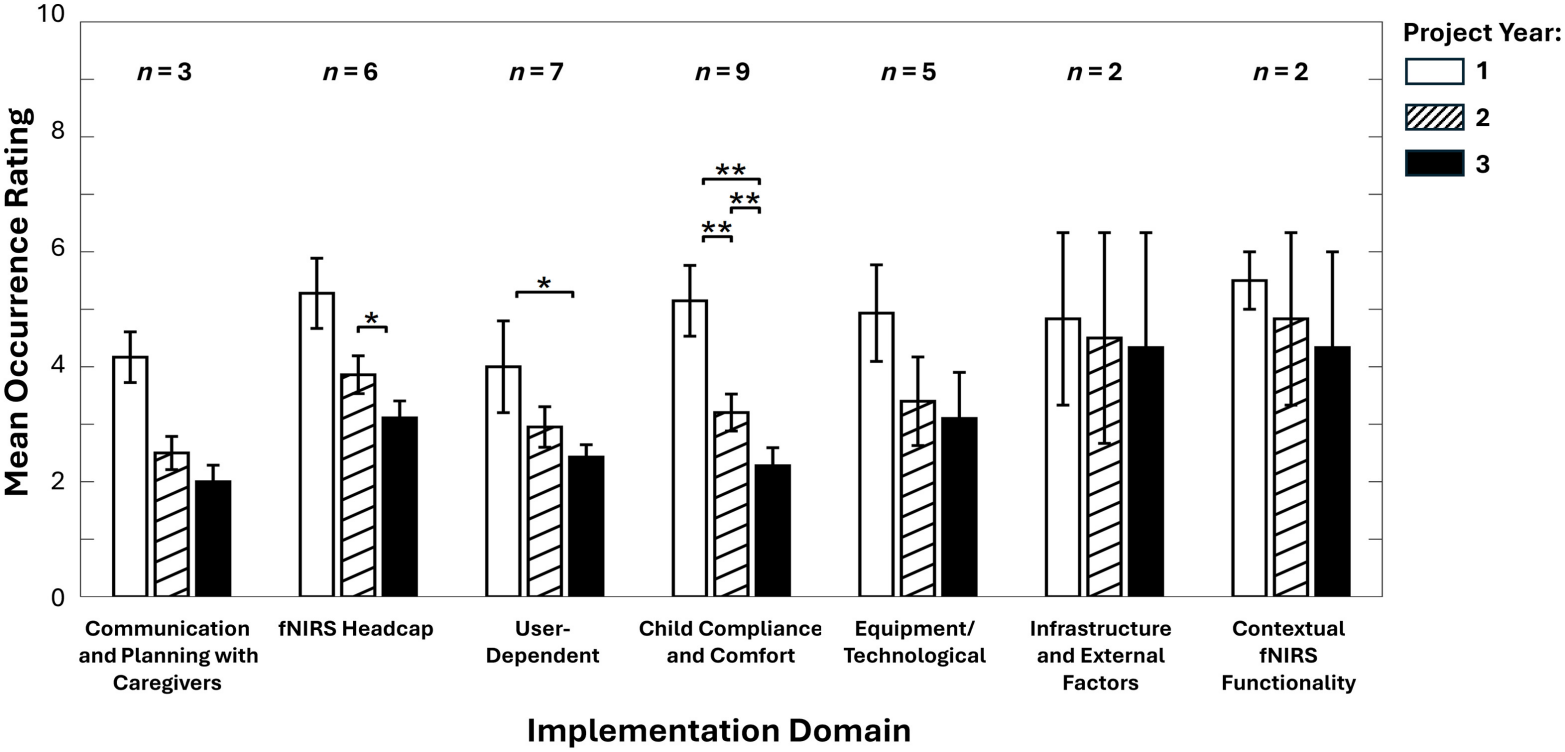
Comparison of yearly occurrence ratings within each implementation domain. Of the total 47 challenges identified for the study, 32 were rated separately by year and are shown here. Ratings were averaged within each implementation domain for project years 1, 2, and 3. Error bars represent standard error. The number of challenges included per domain is displayed as “n =.” Significant effects from pairwise comparisons among project year ratings are marked with asterisks: *p≤0.05 and **p≤0.01.

Figure 3 shows the occurrence ratings for the 32 challenges rated separately for each project year, displaying the averages for each year and within each implementation domain. For every implementation domain, the highest average occurrence rating was in the project year 1, followed by year 2 then year 3. In the Child Compliance and Comfort domain, occurrence ratings significantly decreased from years 1 to 2 (p=0.008), from years 1 to 3 (p=0.004), and from years 2 to 3 (p=0.008), indicating a consistently significant decline over time. In the User-Dependent domain, a significant decrease was observed from years 1 to 3 (p=0.03). Similarly, in the fNIRS Headcap domain, occurrence ratings significantly decreased between years 2 and 3 (p=0.03). No other comparisons reached significance.

### 3.3 Major Challenges to Prioritize

#### 3.3.1 High-risk challenges by RPN

**Fig. 4:**
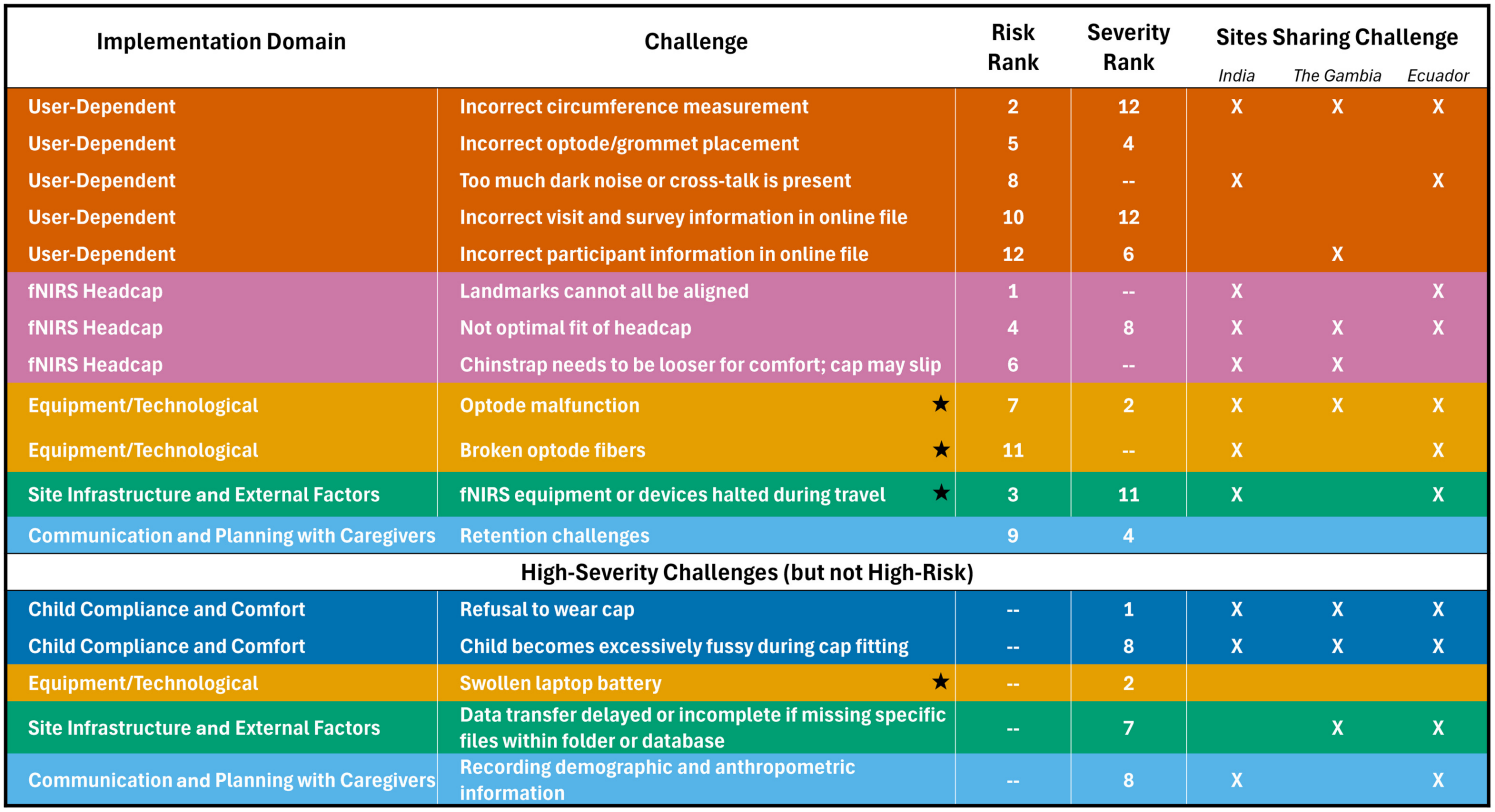
High-risk challenges (identified as those with RPN across the full study duration in the top quartile) and high-severity challenges (identified as those with severity rating in the top quartile). The high-severity challenges that are not classified as high-risk by RPN are at the bottom of the table. Risk Rank indicates comparative RPN, with “1” denoting the highest risk; severity rank indicates comparative severity rating, with “1” denoting the highest severity. Not all high-risk challenges fall into the high-severity classification and vice versa; therefore, rank entry is denoted “--” for non-top quartile ranks. Stars indicate challenges that also fall into the Contextual fNIRS Functionality Implementation Domain. The Sites Sharing Challenge columns indicate whether a challenge was also reported in other projects, based on the poll responses from researchers with fNIRS experience in India,^30^ The Gambia,^27^^,^^28^ and Ecuador.^29^

The RPNs calculated for all 47 challenges ranged from as low as three (for “caregiver wariness when seeing the system”) up to 167 (for “landmarks cannot all be aligned”) (M=46, SE = 6). Twelve challenges were classified as high-risk due to their RPN values falling in the upper quartile (see top section of Fig. 4). The cutoff for the top quartile of RPNs was 49.9, and the range of RPN values for the 12 high-risk challenges was 50.0 to 166.6. These high-risk challenges occurred across all of the fNIRS study procedures, except for fNIRS data collection (see Fig. 2, with high-risk challenges indicated by solid black borders). Among all procedures, headcap preparation (i.e., procedure 3 in Fig. 2) had the highest number and proportion of high-risk challenges: four out of five identified challenges (80%) were high-risk. Among all implementation domains, User-Dependent had the highest number and proportion of high-risk challenges (five out of nine or 56%). The averaged RPN across all challenges within each implementation domain can be found in Fig. S2 in the Supplementary Material.

#### 3.3.2 High-severity challenges

Challenges were classified as high-severity if the severity rating fell in the top quartile (n=13). The cutoff for the top quartile of severity was 7.4, and the range of severity ratings for the 13 high-severity challenges was from 7.4 to 10. Figure 4 displays the high-severity challenges, also marked by dashed borders in Fig. 2.

As indicated in the lower section of Figs. 4 and 2, five challenges that received high-severity ratings were not classified as high-risk challenges. This discrepancy can be explained by low detection ratings (≤2.0) across all five challenges, indicating a higher likelihood that these challenges would be identified and mitigated. In contrast, all but one of the challenges that ranked in the top quartile of both RPN and severity ratings had detection ratings of 3.5 or higher. The one exception (challenge: “incorrect participant information in online file”) had a detection rating right at the threshold of 2.0 and the lowest occurrence rating (2.0) of the high-risk challenges, indicating it was the high severity rating that resulted in a top-quartile RPN. The occurrence ratings corresponding to challenges with top-quartile severity ratings do not differ meaningfully between those which also are high-risk and those which are not (as indexed by RPN).

The averaged severity ratings across all challenges within each implementation domain can be found in Fig. S3 in the Supplementary Material.

#### 3.3.3 Challenges common to multiple projects and locations

Six of the 47 total challenges identified in the BMT project were also experienced by all 3 locations (India, The Gambia, and Ecuador) of the external projects polled (see Fig. 4 for the 5 which were in high-risk or high-severity categories). The challenges spanned four implementation domains and included: “optode malfunction,” “incorrect circumference measurement,” “not optimal headcap fit due to design limitations,” “refusal to wear cap,” “child becomes excessively fussy during cap fitting,” and “child is fussy” during data collection. All three sites represented in the poll reported a variety of system malfunctions, as well as general electrical issues, which were often related to environmental conditions and requiring replacement of parts or maintenance. Though not specified as a challenge prior to conducting the poll, slow or unstable Internet connection was specifically named by two of the three represented locations and shared by the BMT project. An additional challenge reported, which was specific to the study in The Gambia and which the BMT project did not encounter, was torn headcaps that required replacement.

Of the 12 BMT high-risk challenges, three were also identified in all three locations of the polled sites, five were reported by two sites, and four challenges were not reported by any of the polled sites (Fig. 4). All of the high-risk challenges from the fNIRS Headcap, the Equipment/Technological, and the Site Infrastructure and External Factors implementation domains were experienced by multiple polled project locations.

The mitigation strategies identified by FMEA, poll, and a targeted (non-systematic) literature search of developmental fNIRS studies conducted in LMICs^17^^–^^19^^,^^26^ were compiled into a comprehensive table reporting all effective mitigations (see Table S1 and Results S3 in the Supplementary Material).

## 4 Discussion

In this work, we used FMEA to systematically identify the major challenges encountered during a longitudinal developmental fNIRS project in the Mirpur area of Dhaka, Bangladesh, a densely populated region characterized by high levels of poverty. FMEA revealed major challenges, some of which align with those reported in prior studies, whereas others extend beyond previously documented issues. In the following discussion sections, we discuss our findings in the context of high-risk and high-severity challenges, cross-site comparisons and the utility of the FMEA framework, general recommendations, and study limitations.

### 4.1 Major Challenges Encountered in Dhaka (BMT)

#### 4.1.1 High-risk challenges

The FMEA process identified 47 challenges across all fNIRS relevant procedures of the project (see Fig. 2). Of these, 12 were rated as high-risk due to their potential to compromise data quality and integrity (see also Fig. 4). Most of these high-risk challenges fell into three key domains that warrant particular attention: fNIRS Headcap fit, User-Dependent (experimenter) errors, and Contextual fNIRS Functionality (i.e., fNIRS system performance problems related to infrastructure and environmental conditions; see Table 1 and Fig. 4). Note that, when all 47 challenges were grouped by domain, the highest average RPN scores were observed in these three domains, corroborating their relevance (see Fig. S2 in the Supplementary Material). The headcap’s design did not accommodate the full range of head shapes in the BMT project population. This issue is more likely to occur in non-Western contexts, where headcap designs for local common head shapes are still limited. In BMT, the headcap size matching the circumference measurement did not always accommodate head height. Using a larger headcap size improved fit for some children, but not all. Not optimal headcap fit led to inconsistent scalp, and therefore cortical, coverage across participants; it also caused child discomfort and restlessness, increasing motion artifacts in the fNIRS recordings. These issues ultimately reduced data quality and statistical power. Similarly, limited familiarity with study protocols and the fast pace of data collection (up to five visits per day in the BMT project) contributed to experimenter (user) errors. Testing schedules with numerous daily visits, such as the one applied in the BMT, may also occur in other research contexts; however, such schedules are particularly common in research projects conducted in LMICs, where large samples are often assessed longitudinally using a variety of approaches (e.g., neuroimaging, behavioral, and clinical) with fNIRS being one method (e.g., the BRIGHT and BRIGHT kids projects in The Gambia, which followed ∼200 children from infancy to toddlerhood^17^^,^^32^). In the BMT project, tight scheduling further resulted from the requirements of the clinical trial component. Additional constraints that might be more prevalent in projects taking place in LMICs, such as limited testing space and fewer opportunities to train study personnel, can further complicate the management of high-volume data collection and potentially increase the risk of human error. The high-risk mistakes in the BMT included incorrect head measurements and headcap preparation, all of which led to similar consequences as headcap fit issues. Additional experimenter errors, such as incorrect documentation of visit details or participant information, increased the risk of missing or unusable data. Context-specific factors (e.g., power outages, dust, humidity, and customs delays in delivering fNIRS equipment) impacted fNIRS system performance and study timelines. These interruptions sometimes caused participants to miss their testing window, thus reducing sample sizes and weakening longitudinal comparisons. An additional high-risk challenge involved participant retention (domain: Communication and Planning with Caregivers), emphasizing the importance of effective coordination with caregivers.

#### 4.1.2 High-severity challenges

Beyond these high-risk challenges, we identified five high-severity challenges (see Figs. 2 and 4). High-severity challenges are defined as those that can substantially disrupt data collection or compromise data quality when they occur, regardless of how frequently they occur. These challenges were readily detected and addressed by the team but could have caused major disruptions if left unmanaged. For instance, a swollen laptop battery (Equipment/Technological domain and Contextual fNIRS Functionality domain) could have affected equipment functionality and safety, whereas missing files during data transfer (Site Infrastructure) and errors in parent-reported data (Communication with Caregivers) posed risks of data loss or reduced validity. Two high-severity challenges were tied to child behavior: refusal to wear the headcap and excessive fussiness during cap fitting. Although common in developmental research across all contexts, such issues can still be critical, especially for longitudinal designs, and hence are important to monitor.

Together, these findings highlight the main areas of concern and point to specific challenges that require targeted mitigation strategies in the Dhaka-based project and suggest that even well-managed challenges require ongoing oversight, particularly in complex LMIC study environments.

### 4.2 Comparison with Other Sites and Benefits of FMEA Framework

Our findings are also applicable to developmental fNIRS research in a range of other LMIC contexts. Notably, many of the high-risk and high-severity challenges we identified were also reported by other sites (see Fig. 4), indicating that these are not unique to Dhaka but are likely common across several low-resource settings. This underscores the importance of considering such challenges during the early planning phases of similar projects.

At the same time, our structured FMEA framework enabled us to identify three additional major challenges, classified as both high-risk and high-severity, that were not reported by other studies in our survey and are therefore more specific to the BMT project (see Figs. 2 and 4). These include two challenges linked to experimenter errors (i.e., mistakes in populating headcap with NIRS optodes; errors in reporting visit and survey information in online file), and one to planning and coordinating with caregivers (i.e., retention difficulties due to family moving out of study area; discomfort in past neuroimaging sessions; duration of project visits). This illustrates the value of FMEA in detecting underrecognized or project-specific issues that may otherwise be overlooked, thus extending previous work by highlighting additional relevant challenges to consider when implementing fNIRS study in LMICs. Unlike previous descriptive reports, our analysis systematically assessed challenges in all study phases, incorporating input from team members with diverse expertise (e.g., fNIRS data collection, management, and analysis; see Sec. 2.2.1), allowing for a more comprehensive identification and understanding of challenges as well as associated risk.

Furthermore, compared with previous reports, we were able to examine how the frequency of challenges changed over time. Most fNIRS-related issues became less common as the project progressed (see Fig. 3). Recall our longitudinal study followed children from 1 to 3 years of age; with 1 year olds primarily tested in year 1. The decline in occurrence can be attributed to several factors, including developmental changes in children (e.g., longer attention spans, reduced need for breaks, and greater comfort with the project setting) in combination with improved headcap fitting due to overall growth (e.g., headcaps fit 2 and 3 year olds better, and chinstraps were easier to secure with longer necks at these ages). It also reflects increased staff experience with study protocols, technological issues, and managing child behavior. Thus, these findings suggest that some challenges can be reduced over time. Identifying and addressing these challenges, potentially using FMEA, early in the study would be important to reducing their negative impact.

Although we used FMEA retrospectively, applying it prospectively or during ongoing project activities (as is more common in FMEA literature) could support implementation, especially in complex or time-sensitive designs such as longitudinal or multimodal intervention projects. For example, applying FMEA during a project planning phase can serve as a structured design review to support study initiation, staff training, and workflow protocols. In ongoing projects, FMEA can be adapted and simplified for use in regular team meetings, where relevant team members can report emerging issues and record mitigation strategies in a shared log to facilitate timely responses. Regardless of the project stage at which FMEA is applied, its effective use requires involving relevant experts. As done in our FMEA implementation, these experts should have familiarity with the study procedures and with the socio-cultural context of the data collection site, as well as first-hand experience in participant recruitment, data collection, device operation, and data processing. Careful selection of the expert team is crucial to ensure that all aspects of study implementation are adequately covered and to provide appropriate support to the study personnel at every level of the study. In our FMEA application, the project benefited not only from experts familiar with those implementation aspects but also from a staff member whose socio-cultural background spanned both study sites (Bangladesh and the United States). Although not formally included as an FMEA expert responsible for identifying or rating challenges, this staff member played an essential role in facilitating communication between the two sites. Their bilingual fluency and cultural familiarity improved the accuracy and completeness of the FMEA process. Alternatively, we recommend involving an individual with expertise in fNIRS data collection and/or processing who is based at the data collection site. When neither of these options is feasible, periodic site visits by such experts, for refresher trainings and direct observation, may help identify and incorporate challenges into FMEA which may not arise during regular online meetings but only become apparent during on-site visits.

Our work serves as proof of concept for applying FMEA to detect and estimate the risk of challenges and inform mitigation strategies in similarly complex research designs. Resources are available to support implementation.^20^^,^^23^^,^^24^

### 4.3 General Recommendations

A key objective of this work was to compile effective mitigation strategies from the projects conducted in Dhaka (BMT and BEAN) and other fNIRS developmental studies in LMICs. A comprehensive collection of mitigations is provided in Table S1 and Results S3 in the Supplementary Material) and includes both established practices commonly used in developmental research across settings, as well as additional strategies that may be particularly relevant for addressing context-specific challenges. Here, we highlight broader strategies that were effective in addressing the high-risk challenges/domains identified in this work and that may be widely applicable. Lastly, here, we identify an area requiring improvement which is crucial for infant developmental studies, particularly in LMICs.

#### 4.3.1 Training

Training was central to addressing User-Dependent errors (e.g., training on uploading data to shared online servers), fNIRS Headcap fitting issues (e.g., adjustment techniques when the headcap does not adapt well to head shape), and Contextual fNIRS Functionality (e.g., minor repairs and system monitoring). In the absence of an onsite fNIRS expert and/or technical staff able to perform equipment repairs (conditions that may be more common in LMIC settings), frequent training sessions and regular data quality checks are critical for timely identification of potential malfunctions or incorrect application of study protocols. Site-specific training also addressed management of the testing environment conditions. This included daily removal of dust from equipment and cables due to high dust levels in urban Dhaka, as well as the proper and continuous use of a dehumidifier in response to high humidity. Additional training sessions addressed the implementation of standardized researcher–child interaction procedures that ensured consistency across sessions while remaining culturally appropriate for the study setting. Environmental and cultural characteristics of the study site must be carefully evaluated when planning training for onsite personnel. When training is provided by team members who are external to or unfamiliar with the local cultural context, it is essential to involve the onsite team in decisions regarding protocol finalization, particularly those concerning interactions with children and families. We noted in Secs. 2.2.1 and 4.2 that in the FMEA team, we included personnel who facilitated cross-cultural communication among study sites. This staff member, whose linguistic and cultural background spanned both contexts (Boston, United States: where data management and methods experts were based; Dhaka, Bangladesh: study site), was also involved in the BMT project team. Their participation enhanced the quality and effectiveness of training delivered by non-local personnel to local, onsite staff. In the BMT, both onsite and online trainings were provided to local personnel; however, onsite training proved especially effective for fostering collaboration and problem-solving.

#### 4.3.2 Routine Equipment Checks

Routine equipment checks are also important, as environmental factors can speed up wear and degradation (domain: Contextual fNIRS Functionality). Although the BMT setup included several mitigation strategies to overcome technological issues due to climate-related factors and infrastructure characteristic (such as AC, dehumidifier, air purifier, and power surges), we still faced seral technological problems. Hence, ongoing monitoring and timely maintenance remain necessary throughout the study period (more detailed suggestions are provided in Table S1 in the Supplementary Material).

#### 4.3.3 Spare Parts

Collecting extra spare parts for the fNIRS setup is also recommended, especially prior to the project start if feasible, particularly in contexts where customs delays can hinder timely replacements.

#### 4.3.4 Establish Trust

Building and maintaining trust with families is key. Although not unique to fNIRS studies, this is particularly essential for successful recruitment and follow up in developmental research. Particularly in Dhaka, the local team’s strong community ties and clear communication about the project and equipment safety supported recruitment and retention (domain: planning and communication with caregivers). For instance, for families who were concerned about potential harm from the fNIRS device, live demonstration from the team (wearing the equipment on themselves) and shared positive community experiences played a critical role in supporting participation. Generally, understanding local cultural and social norms are key to respectful and effective engagement.

#### 4.3.5 Headcap Design

Improvements in headcap design are clearly needed, given that headcap-related issues were among the highest-risk challenges identified. We recommend that manufacturers improve headcap material and design to better accommodate diverse head shapes, develop more flexible and age-adaptable headcaps, and create softer, more comfortable chinstraps to ensure secure and gentle placement. These enhancements could significantly improve fit, comfort, and data quality in developmental research. In addition, improved headcap design may mitigate inconsistent optode performance across hair type.^33^^–^^35^ However, researchers should still verify headcap fit for the target population during pilot phases, before broader implementation.

### 4.4 Limitations

Several limitations should be considered when interpreting our findings. The FMEA was conducted in Dhaka and identified challenges and their risk levels may vary in other LMICs due to differences in infrastructure, culture, and resources. In addition, some equipment-related challenges may reflect characteristics of the data-collection instrumentation used in BMT and its interaction with the local environment. As such, other projects may face unique issues or assign different risk ratings, so we caution against generalizing our results without considering nuances of local context and equipment aspects. For example, regarding age-related fNIRS implementation challenges, the young children of the BMT study often had short or shaved hair; in the case of longer or more dense hair with older study populations, there are a different set of necessary accommodations and improvements of fNIRS use and design.^33^^–^^35^

Lastly, RPN scoring is inherently subjective and may vary with team composition. To reduce bias, we included team members with different expertise and treated RPN scores as a guide rather than an absolute measure.

## 5 Conclusion

In conclusion, we successfully applied FMEA to systematically identify key challenges in conducting a longitudinal developmental fNIRS study in Dhaka, Bangladesh. We identified 47 challenges across all project procedures (or phases), with the most critical related to headcap fit, user-dependent factors, and adapting fNIRS technology to the specific resource-limited setting. Although many of these issues have also been reported in other developmental studies in LMICs, our FMEA not only provided further quantitative characterization of those previously reported issues, but it also uncovered additional, underrecognized or project-specific challenges, highlighting the tool’s value in identifying overlooked risks. Drawing on these findings and related research, our work provides practical mitigation strategies to support future studies in similar contexts. This work underscores FMEA’s value in detecting and managing high-risk challenges and ultimately strengthens neurodevelopmental fNIRS research implementation in diverse settings.

## Supplementary Material

10.1117/1.NPh.13.S1.S13011.s01

## Data Availability

The FMEA and poll data used in this work can be provided upon request.
