# Supplementary material for: fNIRS in global child health research: insights from failure mode and effects analysis on a longitudinal project in Bangladesh
Source: Neurophotonics. 2026 Mar 31;13(Suppl 1):S13011. doi: 10.1117/1.NPh.13.S1.S13011 (PMC13038603; doi:10.1117/1.NPh.13.S1.S13011)
Supplement: Supplementary file 1 [file NPh_013_S13011_SD001.pdf]

SUPPLEMENTARY MATERIALS for

fNIRS in Global Child Health Research: Insights from Failure Mode and Effects Analysis on a Longitudinal Project in Bangladesh

Methods S1. Recruitment procedures for the Bangladesh Malnutrition Trial (BMT)

Participant recruitment for the BMT project started in February 2020 with a door-to-door census of approximately 100,000 households that identified 5,736 children aged 11–13 months and 2,314 children aged 34–38 months. Verbal consent was obtained from their guardians to measure mid-upper arm circumference (MUAC) as an initial screening criterion. Children who met the inclusion criteria were invited to the project clinic for further assessment[25]. During the first visit to the clinic, parental consent was obtained, and the child's anthropometric measurements were collected (i.e., weight, height, head circumference, and MUAC). Following World Health Organization standards, weight-for-length/height z-scores (WHZ) were calculated to assess nutritional/growth status. Based on these scores, 159 one-year-old children with moderate acute malnutrition (MAM; WHZ<-2 and ≥-3) were enrolled in the intervention group, 75 one-year-old healthy children were enrolled as the well-nourished comparison group (WHZ>-1), and 75 three-year-old MAM children were enrolled as the outcome reference group. Children in the intervention cohort were randomized to receive either a locally produced or an enhanced supplementary food as the nutrition component of the intervention, combined with an identical biweekly psychosocial stimulation program delivered to both intervention groups. For full details on inclusion criteria and intervention plan see Shama *et al.*[25]

The project was registered with ClinicalTrials.gov (NCT05629624) and received ethical approval from the Research Review Committee (RRC) and the Ethical Review Committee (ERC) of the International Centre for Diarrheal Disease Research, Bangladesh (icddr,b), as well as the Institutional Review Board (IRB) of Boston Children's Hospital, USA, under protocol number PR-21084.

Methods S2. fNIRS Data Collection for the BMT

Prior to each functional Near-Infrared Spectroscopy (fNIRS) session, the first experimenter (a member of the onsite staff) measured the child's head circumference and tested three headcap sizes: the recommended size based on the measurement, one size smaller, and one size larger. This was done to identify the best-fitting cap, as the recommended size did not always provide the optimal fit for each child's head shape. After choosing the headcap, a second experimenter populated the headcap with fNIRS optodes in the array depicted in Figure S1. Next, the first experimenter fitted the headcap (with optodes inserted) on the child's head. To ensure consistency in headcap placement, experimenters ensured that a few reference optodes were aligned to specific scalp landmarks (T7, T8, Fz, international 10-20 system). Once the headcap was well secured,

signal quality was checked via the fNIRS data acquisition software, and photographs were taken of the headcap placement before and after the session.

During the fNIRS session, participants sat on their parent's lap, approximately 1 m away from the stimuli presentation screen. Auditory stimuli were presented via two speakers placed on either side of the screen. The fNIRS session included 3 paradigms: a resting state task (during which a screensaver was displayed to calm the child), an auditory task (also during which a screensaver was displayed to calm the child), as well as a working memory task with visual stimuli to which the child was asked to attend. Each paradigm was approximately 7-10 minutes long, but they were stopped earlier if the child became too fussy or distracted. The parent was asked to interact with the child only when they grew fussy. The second experimenter stood behind the parent and helped redirect the child's attention towards the screen when the child was too distracted. Breaks between paradigms were taken when necessary. Each session was recorded via a video camera placed below the stimuli presentation screen to allow offline coding of the child's behavior (see Figure 1).

Following session completion, families were escorted out, and data were shared with the Boston team (the data analysis project staff) using REDCap External and Dropbox (for video and file transfers). All data transfers were quality checked by the Boston-based data analysis staff to ensure accuracy and reliability.

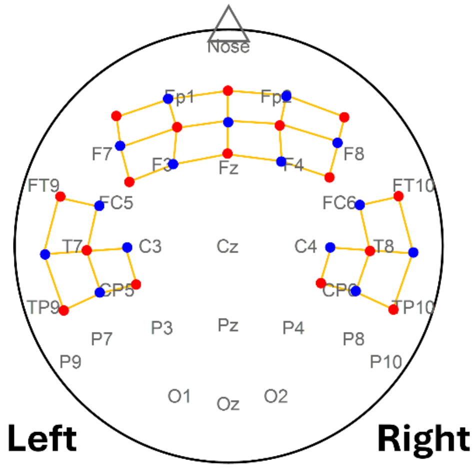

Fig. S1 Optode layout for the fNIRS system used in the BMT project shown from a superior view in 2D. 16 sources (red dots) and 15 detectors (blue dots) for a total of 42 channels (yellow lines).

Methods S3. FMEA Rating Instructions

The following instructions were provided in email and then when meeting as a team prior to rating. Note that "step" in the following instructions corresponds to the sequential "procedures" in Figure 3, and "challenges" in the following instructions hold the same meaning of "challenges" in the main text and shown in Figure 3.

“(Occurrence) Likelihood to happen (1-10)
- How many times, out of 10, do you think this happened?
- *For example*: When trying to schedule a subject during the recruitment phase, how often did you face challenges, regardless of what was causing the challenge?

(Detection) Likelihood it WILL NOT be detected (1-10)
- How many times, out of 10, do you think this challenge may have happened without it being noticed & prevented?
- *For example*: If the fNIRS system had broken fibers, did this go unnoticed when preparing the cap? 10 means unnoticed every time, 1 means you could tell every time in a way that, with the right resources, the issue could be fixed and not affect the rest of the session.

(Severity) Likelihood to cause severe harm or impact on subjects or ability to collect meaningful data (1-10)
- How badly on a scale from 1-10 (10 being the worst) would the occurrence of this challenge seriously affect the subjects' well-being? the systems/devices? the quality/usability of data for analysis?
- *For example*: If a child becomes excessively distressed during initial cap placement, to what degree does this lead to harm to the child? How likely was it the issue would lead to inability to collect data, or unusable data due to very poor quality?”

Methods S4. FMEA Ratings Consensus Procedure

To reach consensus on the averaged ratings, we used a two-step approach. For ratings whose standard deviation was less than or equal to 2.0, the average was accepted as the final rating value. For ratings whose standard deviation was greater than 2.0, the team of experts met to discuss the origin of the discrepancy in the individual ratings (e.g., misinterpretation of rating system, difference in interpretation among experts of the challenge or its effects). Experts could then revise or keep their original ratings, after which a new average was calculated. Due to scheduling constraints and time zone differences between Dhaka and Boston, consensus discussions occurred over multiple online meetings with different team sub-groups. The team member overseeing FMEA instructions, data collection, and ratings evaluation attended all meetings.

Results S1. Rating and Consensus Process

On average, four of the six team experts rated each challenge for detection, five for severity, and three for each set of rating of frequency of occurrence (for each project year and for the overall project duration). Each challenge received ratings from at least two and up to all six experts. The full process of identifying challenges, assigning ratings, and reaching consensus was completed within six weeks.

A total of 50 ratings (15 occurrence, eight detection, 27 severity) out of 204 initial ratings exceeded a standard deviation of 2.0 and required a team discussion before re-rating and reaching the final score. One round of consensus discussion and re-ratings was performed, after which the standard deviation of the newly calculated ratings no longer exceeded 2.0. Among the occurrence ratings requiring discussion, four of the ratings covered the full project period, seven were specific to Year 1, two to Year 2, and two to Year 3.

All detection and severity ratings of the 47 challenges were assigned once for all study years, and the averages were 2.3 (SE = 0.2) and 5.8 (SE = 0.3), respectively.

Results S2. Comparison Between Domains of RPNs and Severity Ratings (all 47 challenges included)

Figure S2 shows the average (*SE*) RPN values per implementation domain. The fNIRS Headcap domain had the highest average score, followed by the User-Dependent domain, then followed closely the Contextual fNIRS Functionality domain. The Child Compliance and Comfort domain had the lowest average RPN. The RPNs were significantly different when comparing the User-Dependent domain to the Child Compliance and Comfort domain (p = .02) and to the Communication and Planning with Caregivers domain (p = .04).

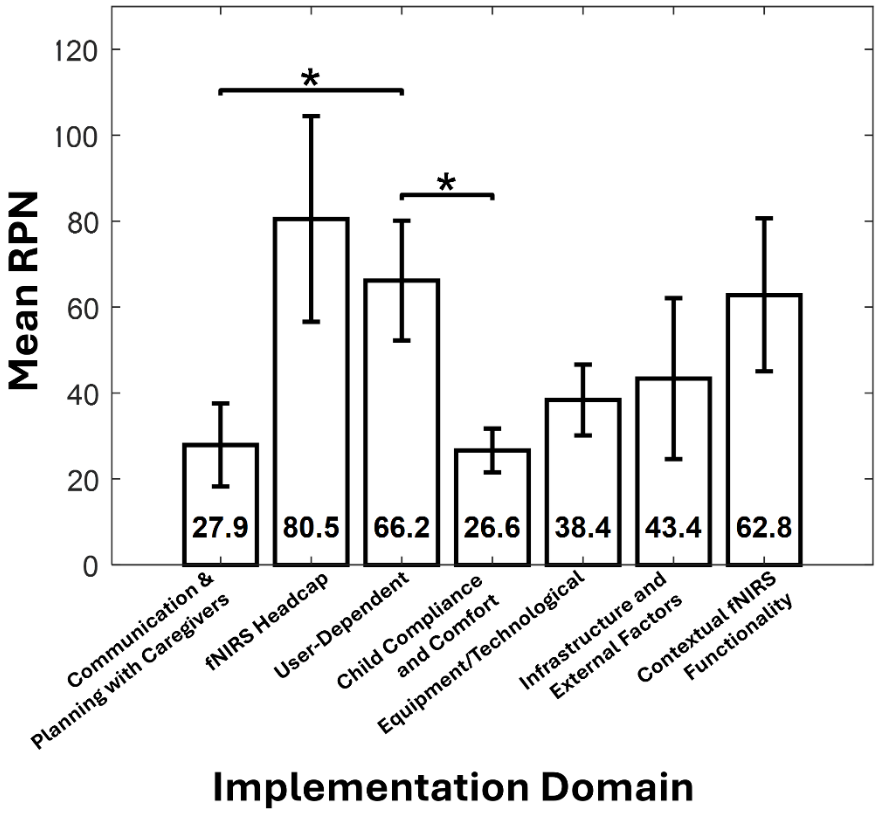

Fig. S2 Comparison of Risk Priority Numbers (RPNs) across implementation domains. All 47 challenges are represented. Per implementation domain, the RPN values from challenges classified into the domain were averaged. Error bars represent standard error. Welch's t-test (two-sample, unequal variances) was performed between domains; the asterisk indicates significant difference ($p \leq .05$) between implementation domains' RPNs.

The severity ratings averaged per implementation domain (Figure S3) reveal no notable patterns.

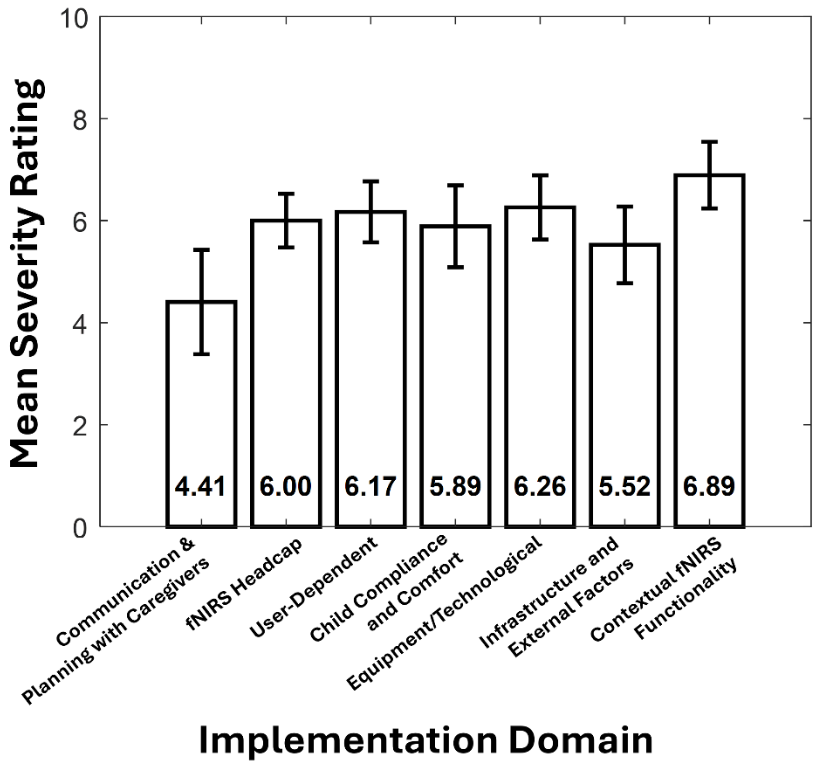

Fig. S3 Comparison of severity ratings across implementation domains. All 47 challenges are represented. Per implementation domain, the severity rating from challenges classified into the domain were averaged. Error bars represent standard error. Welch's t-test (two-sample, unequal variance) was performed between domains.

Results S3. Best Practices and Suggested Mitigation Strategies

When evaluating the list of identified challenges in BMT project, we noted that some of the possible causes, effects, and mitigation strategies repeated across multiple challenges. For example, a possible cause identified for several challenges was environmental conditions (humidity, temperature, dust, etc.), an effect that appeared across 10 challenges was 'delays in testing/data collection', and a mitigation strategy common to 10 challenges was provision of additional or frequent 'training' or 'feedback'. Rather than presenting strategies for each challenge individually, we grouped overlapping elements into broader "issues" (e.g., those linked to high humidity, high temperatures, and dust). This allowed us to reduce repetition and highlight practical strategies identified across BMT, BEAN, and additional studies conducted in LMICs (see Methods section 2.4 for more details on how effective strategies were collected). Recommended Mitigation Strategies are reported in Supplementary Table S1. This resulted in 24 'issues', with a total of 106 mitigation strategies (on average per issue, four) that have been practiced by one or more groups. Fourteen mitigation strategies were reported as prospective, i.e., recommended as future advances or potential solutions; five of these may be addressed by manufacturers.

Table S1: Mitigation Strategies compiled from FMEA, relevant literature [17-19,26], and a poll of researchers[27-30] with experience in projects similar to BMT. Recommended (from experience) and prospective (not reported as implemented) strategies for 24 encountered issues span across implementation domains. Note that to provide a mitigation-focused summary of our results, we grouped related challenges and their causes into broader 'issues' based on shared mitigation strategies (described in Supplementary Results S3). For each issue, we list the effective strategies used in BMT and other projects. Asterisks indicate strategies used by other sites only, and not by the BMT project.

Implementation Domain	Encountered Issues (Dhaka)	Recommended Mitigation Strategies (Dhaka, and Other sites*)	Prospective Mitigations or Solutions
Communication & Panning with Caregivers	Caregiver wariness / distrust of technology	• Plan for extra time during visits to explain fNIRS safety<br>• Show photos of fNIRS data collection to families<br>• Share previous participants' positive experiences<br>• Demonstrate fNIRS use on a staff member or the child's caregiver<br>• Offer a group orientation to tour the facility to families interested in the study*	• Develop and provide more engaging educational materials (e.g., videos; book/paper material to bring home)
	Scheduling difficulties	• Adapt by rescheduling cancelled sessions<br>• Adapt by asking families to come in last-minute to fill in slots freed by cancellations (common in Dhaka)<br>• Ensure an additional staff member is available to care for child's sibling(s)<br>• Allow older siblings (over 1 year of age) to quietly sit with caregivers in fNIRS room if an additional staff member unavailable<br>• Use more than one testing room to allow for multiple concurrent sessions<br>• Reduce the family's total time in project site by optimizing flow between fNIRS and other sessions (e.g., behavioral)<br>• Consider religious observances and when scheduling visits	
	Difficulties recording demographic and other information on child and family	• Validate document translations for comprehension<br>• Work with local onsite assistants to create standardized protocols/scripts to ensure clear, consistent communication with families<br>• Create a comfortable, private setting for caregivers to report responses to questionnaires, reassuring them about data deidentification and privacy.<br>• Instruct onsite staff to record responses word-for-word to avoid subjective interpretation<br>• Conduct frequent quality control check-ups on participant information	

	Retention difficulties	• Ensure the expected visit duration is effectively communicated to caregivers prior to the session<br>• Reassure caregivers that fNIRS session requires minimal preparation and is safe<br>• Provide transportation for caregivers and children from their home to the study site and back home<br>• Provide breaks as well as snacks and lunch for caregiver(s) and child(ren)<br>• Maintain continuous engagement and communication with families	
fNIRS Headcap	Headcap shifts during fNIRS session	• Secure the chinstrap more tightly for stable headcap placement (note: less effective on children with shaved heads)	
	Headcap causes itchiness or discomfort to child	In addition to "Child Compliance and Comfort: Child behavior (e.g., fussy, sleepy, hungry)":<br>• To address heat-related discomfort, use air conditioning and/or a fan<br>• Optimal chinstrap tightness to maximize headcap secureness without increasing discomfort	• fNIRS manufacturers: develop improvements in material and design of headcap to relieve discomfort and optimize airflow<br>• Select a headcap which allows airflow to the scalp (e.g. ninjaCap)
	Difficulties in headcap size selection, placement, and adjustment due to designs not matching head shapes	• Provide training to onsite staff on optimal cap placement and on scalp landmark identification<br>• Try various headcaps on the child to find the best for fit and comfort<br>• When ears do not align with the headcap's ear holes, tuck the child's ears under the headcap *<br>• With permission and if applicable, remove child's hair braids prior to fNIRS data collection*<br>• Perform digitization to localize array, rather than solely rely on headcap placement*	• fNIRS manufacturers: develop more flexible and age-adaptable headcaps<br>• fNIRS manufacturers: develop more comfortable and soft chinstraps
User-Dependent	Difficulties in head measurements and headcap placement	• Conduct multiple training sessions, online and onsite, for head circumference measurement and cap selection<br>• Onsite staff may optimize time by measuring the child's head and begin populating optodes into headcap while other staff are consenting, allowing more time for thorough measurement, size selection, and optode population into headcap	

	Incorrect optode/grommet placement onto headcap	• Provide training sessions for onsite staff and technicians<br>• Onsite staff take photos of headcap placement for onsite or data analysis staff to check optode placement offline	
	Poor quality of photos (capturing headcap placement)	• Use a camera with higher resolution and faster shutter speed<br>• Data analysis staff conduct daily quality check-ups and provide feedback to onsite staff<br>• Provide additional training and a detailed protocol on headcap photo process<br>• Alternatively, onsite staff take video recordings of headcap placement	• Develop Software/App providing real-time feedback on headcap fitting and optode localization, which can be used in various light conditions and are optimized for children
	Inconsistency in oral instructions given to caregiver/child	• Minimize instructions given to caregivers for task completion, when applicable<br>• Provide frequent feedback/trainings to onsite staff	
	Incomplete or incorrect data documentation	In addition to "Communication & Planning with caregivers: Recording demographic and other information on child and family," and "Site Infrastructure & External Factors: Incomplete data transfer":<br>• Request onsite staff to send photos of hardcopies of files to data analysis staff<br>• To help troubleshoot any data entry errors, set up tracking in the collection database (i.e. REDCap) to trace when and by whom changes are made (e.g. version history)<br><br><u>For critical participant or testing information:</u><br>• implement onsite validation measures (e.g., collect the same information from multiple sources during the study visit)<br>• measure information multiple times in different occasions (e.g., once during home visit and once during site visit)*	
Child Compliance and Comfort	Child behavior not conducive to data collection (e.g., fussy, sleepy, hungry)	• Reduce weight of fNIRS fibers on child's head by hanging fibers (i.e. on an IV pole) placed behind the child<br>• Provide tools for engaging the child (e.g. toys, bubbles) and train onsite staff to use them at appropriate moments during data collection<br>• Provide soft snacks and breaks before or during fNIRS session<br>• Re-arrange tasks order during lab visit to accommodate child needs (e.g., start with behavioral tasks to help them acclimate to people and the environment) *	• Select wireless and more lightweight fNIRS systems to improve child comfort and movement<br>• fNIRS manufacturers: develop child-friendly session prep kits

		• Adapt by delaying fNIRS assessment (e.g. to allow the child to sleep) or finish the session early *	
	Child scared of dark room	• Install a low-level, controlled light source (i.e. LED light) to softly light the fNIRS room<br>• Perform standard child-soothing methods	
Equipment/ Technological	fNIRS system malfunction	Monitor the system:<br>• Before starting the project, ensure all fNIRS system parts are working properly (especially if it is not a new system)<br>• Before each day of data collection, the onsite staff should ensure all fNIRS system parts are working properly<br>• To recognize any emerging specific channel issues: onsite and data analysis staff monitor channel signal levels across several participants (e.g., unusual noise or patterns in the signal, low-intensity signal)<br>• Onsite staff routinely check on detector functioning to identify broken detectors<br>• Onsite staff frequently check of sources power level to monitor decrease in performance and plan timely replacements*<br><br>Prevent damage:<br>• To help prevent children from grabbing optode fibers, apply a shower cap over the populated headcap (note, this may create more discomfort for some children)<br>For Troubleshooting:<br>• Create protocols for, and provide training in, electrical connections training to troubleshooting equipment for onsite staff<br>• In case of interactions between the onsite staff and non-local fNIRS manufacturer, involve a translator to facilitate effective communication<br>• Schedule fNIRS repairs to align with study and onsite staff availability when possible<br>• See "Site Infrastructure & External Factors: Shipping Regulations" strategies in relation to addressing system repair/arrival delays*	• Project team or other fNIRS users with relevant experience: develop and provide global troubleshooting guide based on shared experiences across sites<br>• fNIRS manufacturers: provide information on system optimal conditions and improve system resistance to or provide assistance coping with certain conditions (e.g., high humidity, dust etc.)
	Other equipment failure (laptop, camera etc.)	• Before initiating the project, purchase new equipment as able and perform equipment check-up on new or existing equipment (e.g., cables, laptops batteries, speakers, monitors)<br>• Throughout the project, onsite staff perform frequent equipment check-ups and replace damaged parts as able<br>• Before each data collection session, restart laptops (e.g. task presentation and fNIRS recording) to avoid loading issues	

	Difficulties optimizing scalp-coupling and signal quality	• Move hair aside from between the optodes and scalp<br>• Use the fNIRS system's light intensity levels self-calibration<br>• In the case that system self-calibration is too time-consuming: per age group, manually set the light to levels that are known to work. Then, as needed, manually adjust individual channel light levels.<br>• Use a shower cap to block ambient light (from presentation laptop, testing space, etc.) from the fNIRS detectors*<br>• Apply a minimal amount of clear hair gel or ultrasound gel to hair that is moved, per optode necessary, to help keep it out of the way*<br>• Use air conditioning and/or a fan to reduce sweat while wearing headgear (sweat induces optode slippage, causing signal shifts)*	
	Breakage of headcap parts	• Order extra parts (e.g. optode grommets/holders, headcaps) from fNIRS manufacturer<br>• Perform custom fixes: make your own replacement parts (e.g. 3D print optode holders), adjust the headcap holes (e.g. sew the cap)	
Site Infrastructure & External Factors	High humidity, temperatures, and dust levels	• Use an air conditioner, air purifier, and dehumidifier in fNIRS room<br>• Clean fNIRS room and dust equipment daily<br>• When not in use, cover fNIRS system with a box (e.g. cardboard) with ventilation holes	• Identify and use equipment built to withstand high temperatures and sweat on scalp (source-detector drift)
	Power outages	• If outage is scheduled (e.g. load-shedding), plan visits before or after power outage<br>• Set-up a power back-up system, voltage stabilizer, surge protector<br>• Provide electrical connections training to the onsite personnel, to enable troubleshooting of power and potential damage	• Use built-in voltage regulators
	Non-constant or bright ambient light levels in fNIRS room	• In the case of windows, block the light (e.g., dark curtains, cardboard, etc.)<br>• Ensure all in-lab light sources are consistent levels across visits and check for interference with fNIRS system<br>• Choose a windowless room for fNIRS data collection, when possible*<br>• For an outdoor testing space, provide shade (e.g. roofing plastic, tent, tarpaulin, etc.)*	
	Shipping regulations	• Prepare appropriate shipping documentation, compliant with local and international regulations (local staff can help)<br>• For delays of equipment arrival: plan for, and adjust to potential delays in	• Identify local manufacturer and supplier partnerships

		equipment arrival when scheduling visits<br>• For delays in replacement or repair: as feasible, purchase extra materials to allow continued data collection<br>• Package fNIRS equipment with extra protection/cushioning when transporting	
	Acoustic noise	• Limit verbal interactions between onsite staff, the caregiver, and the child(ren) to between-task timepoints<br>• During data collection, power off the air conditioning and instead use a quieter fan to minimize noise<br>• Use bubbles or other strategies to re-direct attention of the child to the paradigm when appropriate<br>• During data collection, onsite staff may place themselves outside the fNIRS room to manage and prevent loud talking, doors banging, etc.*	• Soundproof the fNIRS room as much as possible
	Incomplete data transfer	• Set up secured data transfer protocols for PHI information<br>• Back up data on a secondary hard drive (in addition to the data acquisition computers)<br>• To reduce missing data, implement direct electronic data entry with user-friendly portable devices (e.g., larger tablets, larger fonts, simplified record selection) and require all fields<br>• Onsite staff may transfer data weekly, and data analysis staff may perform weekly quality check-ups<br>• Ship encrypted hard drives as a backup*	
Summary of General Good Practices		• Perform comprehensive fNIRS system check-up prior to study start<br>• Perform regular equipment maintenance<br>• Conduct regular data quality check-ups (fNIRS, participant information, etc.)<br>• Provide a kit for small repairs onsite<br>• Provide back-up equipment or consumables when possible<br>• Continuous community engagement<br>• Extensive and repeated training and protocol reviews for onsite staff (consider training different team members as experts in specific project parts)<br>• Develop culturally relevant cognitive tasks<br>• Create culturally appropriate stimuli and paradigms	
